# Complement activated granulocytes can cause autologous tissue destruction in man

**DOI:** 10.1155/S0962935192000279

**Published:** 1992

**Authors:** E. Löhde, H. Raude, M. Lück, E. Kraas, W. Lierse

**Affiliations:** 1 Surgical Department Academic Hospital Moabit Turmstr. 21 Berlin 21 D-1000 Germany; 2 Institut of Neuroanatomy Martinistr 52 University of Hamburg Hamburg 20 2000 Germany

## Abstract

Activation of polymorphonuclear granulocytes (PMNs) by C5a is thought to be important in the pathogenesis of multiple organ failure during sepsis and after trauma. In our experiment exposure of human PMNs to autologous zymosan activated plasma (ZAP) leads to a rapid increase in chemiluminescence. Heating the ZAP at 56^°^C for 30 min did not alter the changes, while untreated plasma induced only baseline activity. The respiratory burst could be completely abolished by decomplementation and preincubation with rabbit antihuman C5a antibodies. Observation of human omentum using electron microscopy showed intravascular aggregation of PMNs, with capillary thrombosis and diapedesis of the cells through endothelial junctions 90 s after exposure to ZAP. PMNs caused disruption of connections between the mesothelial cells. After 4 min the mesothelium was completely destroyed, and connective tissue and fat cells exposed. Native plasma and minimum essential medium did not induce any morphological changes. These data support the concept that C5a activated PMNs can cause endothelial and mesothelial damage in man. Even though a causal relationship between anaphylatoxins and organ failure cannot be proved by these experiments C5a seems to be an important mediator in the pathogenesis of changes induced by severe sepsis and trauma in man.


Mediators of Inflammation, 1, 177–181 (1992)

### Complement activated granulocytes can cause autologous tissue destruction in man

E. Löhde,[1,CA] H. Raude,[1] M. Lück,[1] E. Kraas[1] and W. Lierse[2]

[1]Surgical Department, Academic Hospital Moabit, Turmstr. 21, D-1000 Berlin 21, Germany; [2]Institut of Neuroanatomy, University of Hamburg, Martinistr 52, 2000 Hamburg 20, Germany

[CA] Corresponding Author

**ACTIVATION of polymorphonuclear granulocytes (PMNs) by C5a is thought to be important in the pathogenesis of multiple organ failure during sepsis and after trauma. In our experiment exposure of human PMNs to autologous zymosan activated plasma (ZAP) leads to a rapid increase in chemiluminescence. Heating the ZAP at 56°C for 30 min did not alter the changes, while untreated plasma induced only baseline activity. The respiratory burst could be completely abolished by decomplementation and pre-incubation with rabbit antihuman C5a antibodies. Observation of human omentum using electron microscopy showed intravascular aggregation of PMNs, with capillary thrombosis and diapedesis of the cells through endothelial junctions 90 s after exposure to ZAP. PMNs caused disruption of connections between the mesothelial cells. After 4 min the mesothelium was completely destroyed, and connective tissue and fat cells exposed. Native plasma and minimum essential medium did not induce any morphological changes. These data support the concept that C5a activated PMNs can cause endothelial and mesothelial damage in man. Even though a causal relationship between anaphylatoxins and organ failure cannot be proved by these experiments C5a seems to be an important mediator in the pathogenesis of changes induced by severe sepsis and trauma in man.**

**Key words:** ARDS, C5a, Complement activated granulocytes, Mesothelium, Oxygen free radicals, Sepsis

### Introduction

Systemic complement activation plays a key role in acute organ dysfunction during sepsis.[1–3] It has been demonstrated in several animal models that the lung is one of the main targets of activated complement. Histological changes consist of pulmonary haemorrhage, interstitial oedema, endothelial damage and leucocyte plugging.[4–6]

These changes observed in animal models parallel the findings in patients with severe sepsis as well as after trauma and cardiopulmonary bypass,[2,7] are generally designated as adult respiratory distress syndrome (ARDS) independent of their aetiology. C5a has been considered a potent and important mediator of these changes. Its target is a 47 kD receptor on the cell surface of polymorphonuclear granulocytes (PMNs). C5a mediates the release of free oxygen radicals, adherence, aggregation and chemotactic migration of these cells.[5] These changes may contribute to the development of capillary leakage observed during acute illness.

However, there is a lack of information concerning the actual mechanism of the reaction of PMNs to activated complement in human tissue. For example, it is still unclear whether, and to what extent, the *in vitro* observed C5a mediated release of toxic oxygen radicals causes damage to human cells *in vivo*. Therefore, we used human peritoneum to investigate the effects of activated complement on autologous human tissue.

### Methods

*Cells:* Heparinized blood was obtained from healthy adult volunteers. PMNs were separated by Ficoll density gradient and the remaining red cells lysed with distilled water/1.8% saline solution. PMNs were then washed twice in minimum essential medium (MEM) (Dulbecco pH 7.4 without phenol red and bicarbonate) and resuspended at a concentration of 5–6 × $10^3$ cells/$\mu$l in MEM. Pappenheim staining showed that 98% were PMNs and trypan blue exclusion showed 94–96% of cells to be viable.

*Plasma:* Plasma was obtained from heparinized blood from human volunteers and kept in storage at 4°C for a maximum of 6 h. Complement was activated by incubating the plasma with boiled zymosan (Sigma Chemical Co., St Louis, MO) at 37°C for 30 min followed by centrifugation at 2000 × ***g***. The supernatant was stored at 4°C for a

© 1992 Rapid Communications of Oxford Ltd maximum of 6 h. Decomplemented plasma was prepared by heating the plasma to 56°C for 30 min. C5a inhibition was obtained by preincubation of ZAP with rabbit immunoglobulins cross-reacting to human C5a (DAKO code A O 55 Boehringer-Ingelheim, Germany) at 37°C for 15 min.

*Chemiluminescence:* Chemiluminescence was measured in a Biolumat LB 9500 and LB 922 (Berthold Mod. 210) and data analysis was performed by a Hewlett Packard 97S computer. Isolated PMNs were incubated with Luminol (4-amino-2,3 dihydroxy-1,4-phthalazinedione, Sigma Chemical Co., St Louis, MO) for detection of reactive oxygen compounds. When baseline activity was reached autologous plasma was added to give a final concentration of 32%. The generation of reactive oxygen compounds was continuously measured and computed as the maximal count every 30 s.

*Scanning electron microscopy:* Heparinized blood was obtained from patients scheduled for cholecystectomy and prepared as mentioned above. Part of the greater omentum was carefully removed immediately after opening the abdomen. Tissue specimens were exposed to autologous ZAP and native plasma as well as to MEM with and without the addition of autologous PMN for 0, 1.5, 4, and 25 min. The experiment was commenced within 15 min after surgical removal of the tissue, which was kept humidified throughout. After termination of exposure the specimen was fixed in 2.2% glutaraldehyde followed by osmium exposure. Dehydration was carried out with alcohol in increasing concentrations and exchange to freon. Critical point drying and gold coating was performed in an Edwards sputter coater for 2.5 min and the specimens were evaluated in a Novoscan 30 (Zeiss).

The experiment was approved by the human experimentation committee, and all patients participating gave informed consent.

### Results

Exposure of PMNs to autologous zymosan activated plasma caused a rapid increase of chemiluminescence due to an accelerated generation of free oxygen radicals (Figure 1). The increase could already be detected after 30 s and reached its maximum after 5–7 min. Zymosan activity was preserved even after heating the activated plasma at 56°C for 30 min and the PMN showed an identical pattern of free oxygen generation. Native plasma induced only a baseline activity and decomplementation of the plasma prior to zymosan incubation abolished the generation of plasma activity completely. The respiratory burst of PMN

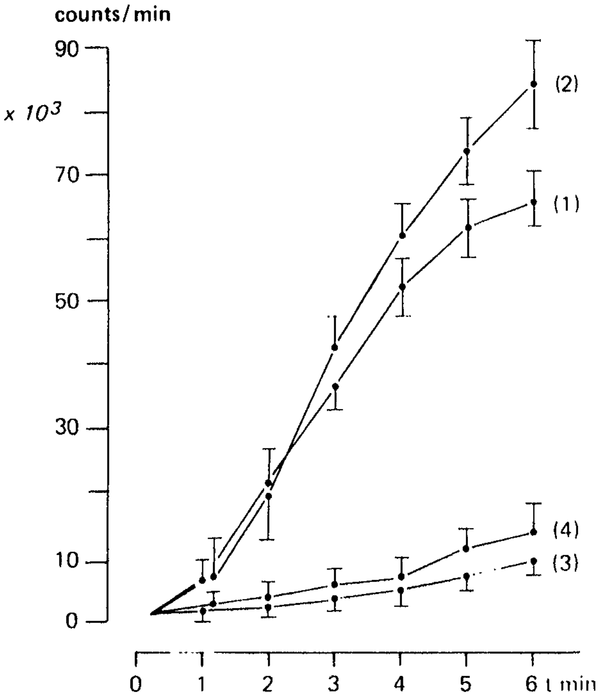

FIG. 1. Chemiluminescence measurement of isolated human PMNs in autologous plasma. The exposure to ZAP (curve 1) as well as heated ZAP (curve 2) induces an increase generation of oxygen derived free radicals from the cells after 30 s. Native plasma (curve 3) does not result in increased metabolic activity. Plasma heating prior to zymosan incubation abolishes the generation of active complement fragments (curve 4).

secondary to exposure to ZAP could be readily inhibited by dose-dependent incubation of ZAP with cross-reacting rabbit anti-human C5 antibodies (Figure 2).

A scanning electromicrograph of the normal greater omentum of man is shown in Figure 3a. The surface is formed by a monolayer of polygonal mesothelial cells with microvilli. They are densely interconnected and overlay connective tissue and fat cells. In addition, numerous superficial vessels can be seen.

Within 90 s after ZAP exposure PMNs appear on the mesothelial surface presumably due to directed migration. Around the PMNs the mesothelial cells retract, the interconnections between these cells break up and gaps appear between the formerly tightly packed cells (Figure 3b).

Four minutes after ZAP exposure the mesothelium in the vicinity of the cells is completely destroyed. The submesothelial connective tissue is now only partially covered by the remaining damaged mesothelial cells (Figure 3c). In these areas even fat cells are exposed while regions more distant to the PMNs remain morphologically intact. Exposure of the omentum to native plasma or MEM does not induce any changes.

A closer examination of the superficial capillaries reveals that diapedisis of PMNs through the vessel wall begins within 90 s after ZAP exposure. Figure

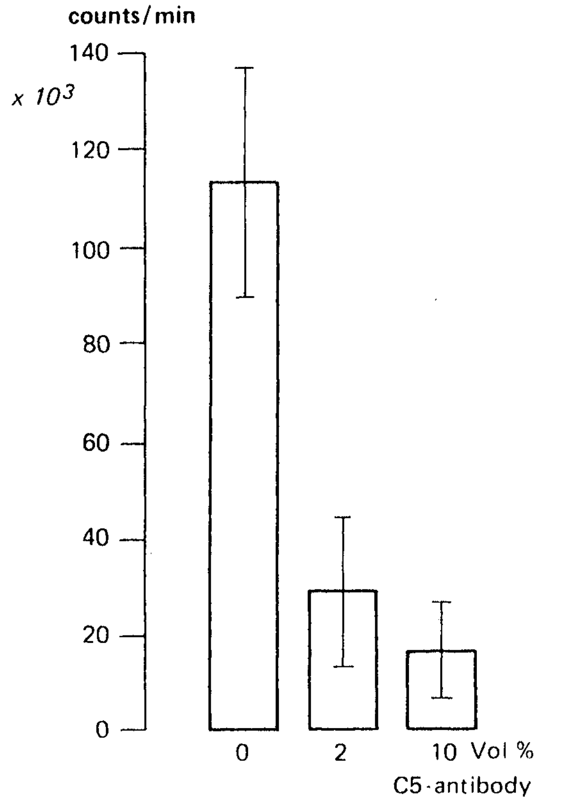

FIG. 2. The ZAP-mediated generation of 02-species from isolated PMNs shown as an integral of the first 4 min after exposure. Prior incubation of ZAP with cross-reacting mouse anti-human C5/C5a antibodies. IgG causes a marked CL-reduction up to 87% of the mean.

4 shows PMNs during the migration through the endothelial junctions of the capillaries. Simultaneously, the formation of intravascular PMN aggregates is observed (Figure 5). The PMNs adhere tightly to each other, the capillary flow is interrupted and the endothelium in the region of the micro-aggregates destroyed. Neither native plasma nor MEM leads to a similar phenomenon.

### Discussion

One essential feature of the interaction of C5a with PMNs seems to be the great speed of reaction. It is due to the small size of the molecule and its high affinity to specific receptors on the surface of PMNs. Exposure to C5a leads to a 50% saturation of the receptors within 1 min.[8]

The data presented here show that the presence of active complement fragments can initiate an activation of PMNs which, in turn, causes intense damage to autologous tissue. After the complement cascade has been activated, foreign antigens (e.g. bacteria) are not necessary for the activation of effector cells. The developing cytotoxicity is undirected but clearly restricted to the immediate environment of the PMNs. More distant tissue areas

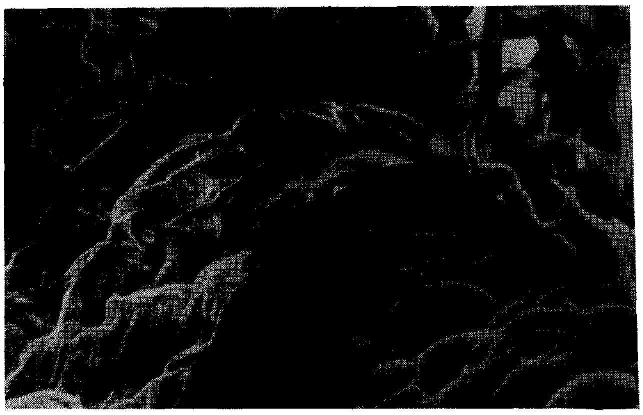

FIG. 3a. Scanning electron microscopy of native human greater omentum. Mesothelial cells form a tight monolayer at the surface. The gross tissue structure is formed by underlying fat cells. Superficial capillaries are demonstrated.

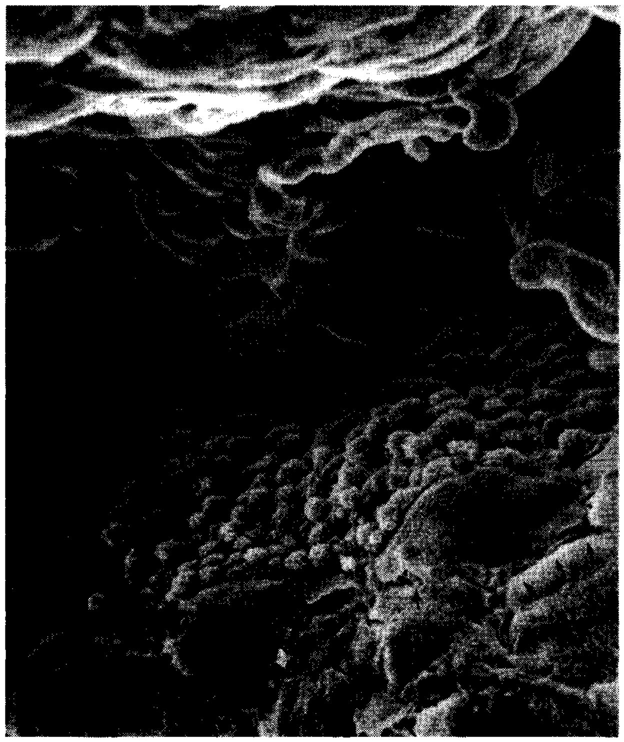

FIG. 3b. 90 s after ZAP exposure. Retraction and disconnection of mesothelial cells in the near environment of activated leucocytes are visible as arrowed. The leucocytes themselves appeared at the tissue surface by their own chemotactic locomotion.

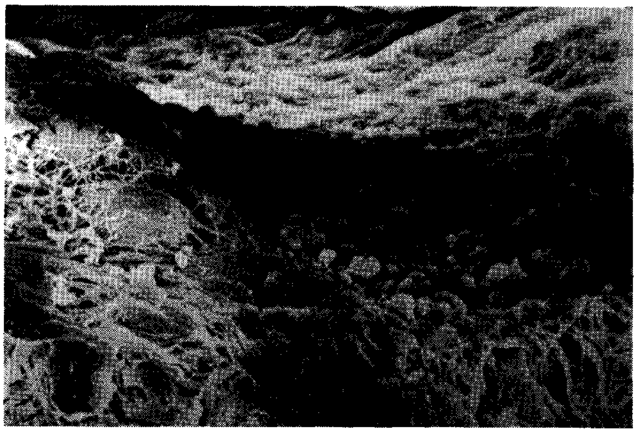

FIG. 3c. 3 min after ZAP exposure. The mesothelial cell layer has been destroyed. The cross network of submesothelial connective tissue and underlying fat cells are uncovered as arrowed.

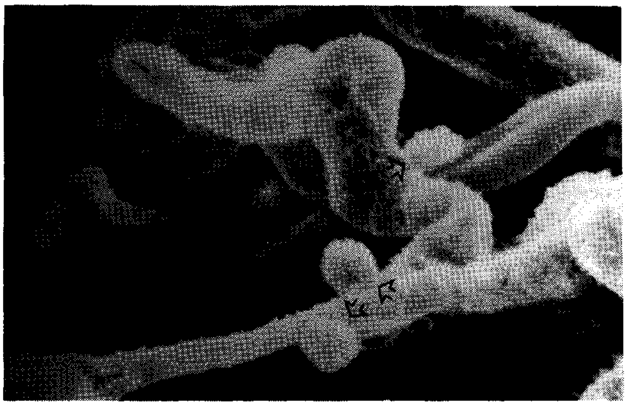

FIG. 4. 90 s after ZAP exposure. Intravascular leucocytes as arrowed have been chemotactically activated and are migrating through endothelial gaps out of the capillaries.

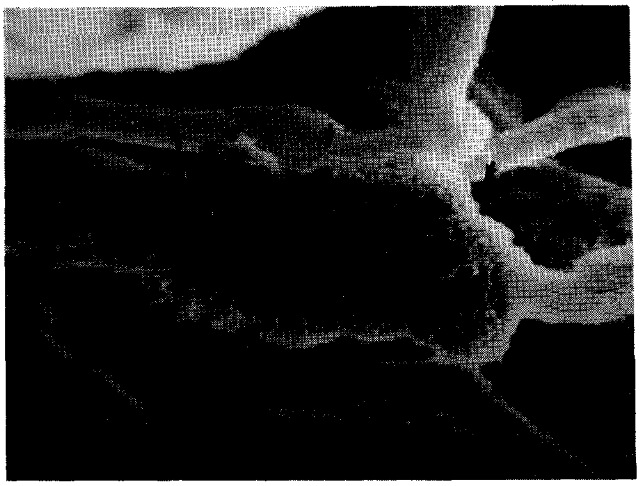

FIG. 5. 90 s after ZAP exposure. Some intravascular activated leucocytes form tight leucoaggregates within the vessels. Subsequently the surrounding endothelium is destroyed and the capillary flow interrupted.

remain morphologically intact. Thus, activated complement does not induce morphologically detectable changes of human mesothelial cells by itself. The damage is rather dependent on the activation of PMNs.

The cytotoxicity of C5a activated PMNs to homologous endothelial cells *in vitro* was first demonstrated by Sacks.[9] The release of free oxygen radicals leads to the oxidation of the unsaturated fatty acids of the phospholipids in the cell wall. Our data also show a close correlation between the mesothelial damage and the kinetics of the oxygen release by PMNs measured by chemiluminescence. A maximal increase of oxygen radical production of isolated PMNs occurred within a few minutes after ZAP exposure. Scanning electron microscopy revealed extensive tissue destruction after the same time interval. A similar temporary relationship was observed by Goldstein *et al.*[10] In their experiments more than 80% of the maximum obtainable free oxygen radicals derived from PMNs stimulated by isolated C5a, as measured by cytochrome reduction, were generated within the first 10 min. The actual intensity of the respiratory burst of PMNs *in vivo* might even be considerably higher because surface contact has been thought to promote oxygen radical release.[11] Data from animal experiments show that the addition of dismutase or catalase serving as oxygen radical scavengers as well as granulocyte depletion can provide significant protection against C5a mediated tissue damage.[6]

The data presented here also confirm earlier observations about the chemotactic migration of PMNs. It is known that C5a as a physiological mediator of inflammation induces chemotactic migration of PMNs.[12,13] However, in spite of the rapid activation of PMNs by C5a a significant effect on directed migration can only be detected after 60 min.[12] Nevertheless, morphological studies of the ZAP activated PMNs reveal the formation of pseudopodia and membrane ruffling within seconds after exposure.[14,15] Other alterations of the cytostructure consistent with the initial phase of directed cellular migration have also been detected within seconds of exposure.[15]

Our data show that the chemotactic response of PMNs to C5a *in vivo* occurs within seconds and follows the same time course as the other C5a related effects. Intervascular PMNs become rapidly activated by C5a and pass through the endothelial junctions of the capillaries into the surrounding tissue. The rapidity of C5a diffusion into the capillaries, binding to the receptors, induction of the cellular response and directed migration out of the vessel emphasizes the potency of this anaphylatoxin *in vivo*.

One of the main morphological features which correlates to the physiological changes of adult respiratory distress syndrome (ARDS) is the formation of microemboli in the pulmonary capillaries by aggregated PMNs. Earlier studies of isolated human PMNs, using electron microscopy, showed the development of tight cell-to-cell aggregations after exposure to ZAP serum.[14,15] Similar functional studies by aggregometry demonstrated a maximal cell aggregation 90 s after ZAP exposure.[14] The mechanism of aggregation is thought to be a reduction of the surface charge of the granulocytes after binding of C5a to receptors.[16]

Our observations confirm that activated complement can lead to the formation of intervascular leucocyte aggregates in human capillaries. Thrombosis of the capillaries could be observed after 90 s, the endothelium was destroyed and vascular flow interrupted. Thus, not only human mesothelium but also human endothelium is susceptible to the C5a induced cytotoxicity of PMNs. The consequences of these changes in capillary flow are transudation, haemorrhage and hypoxaemia. The microvascular structure of the lungs is considered especially vulnerable to the cytotoxic effect of cellular aggregates.[4,5] In fact only 10–15 cells seem to be sufficient for the creation of microvascular destruction within seconds, explaining the rapid physiological effect of leucocyte sequestration caused by systemic complement activation in man.

In summary, the findings presented may well support the concept that C5a activated PMNs play a key role in the pathogenesis of capillary damage in man as it is found in ARDS. Because of our lack of knowledge concerning the regulatory mechanisms of the inflammatory reseponse it would be premature to interpret the data as proving a causal relationship between anaphylatoxins and organ failure. Nevertheless, the complement derived cleavage product C5a can be considered a highly active principle in the pathogenesis of the changes induced by severe sepsis and trauma in man.

### References

1. Hammerschmidt DE, Weaver LJ, Hudson LD, Craddock PR, Jacob HS. Association of complement activation and elevated plasma-C5a with adult respiratory distress syndrome. *Lancet* 1980; **1**: 947–949.
2. Jacob HS. Role of complement and granulocytes in septic shock. *Acta Chir Scand Suppl* 1980; **499**: 97–106.
3. Solomkin JS, Cotta LA, Satoh PS, Hurst JM, Nelson RD. Complement activation and clearance in acute illness and injury: evidence for C5a as a cell-directed mediator of the adult respiratory distress syndrome in man. *Surgery* 1985; **97**: 668–677.
4. Hohn DC, Meyers AJ, Gherini ST, Beckmann A, Markinson RE, Churg AM. Production of acute pulmonary injury by leukocytes and activated complement. *Surgery* 1980; **88**: 48–58.
5. Hosea S, Brown E, Hammer C, Frank M. Role of complement activation in a model of adult respiratory distress syndrome. *J Clin Invest* 1980; **66**: 375–382.
6. Till GO, Johnson KJ, Kunkel R, Ward PA. Intravascular activation of complement and acute lung injury—dependency on neutrophils and toxic oxygen metabolites. *J Clin Invest* 1982; **69**: 1126–1135.
7. Jacob HS. Complement-mediated leucoembolization: a mechanism of tissue damage during extracorporeal perfusions, myocardial infarction and in shock—a review. *Q J Med New Series LII* 1983; **207**: 289–296.
8. Chenoweth DE, Hugli TE. Human C5a and C5a analogs as probes of the neutrophil C5a receptor. *Molecular Immunol* 1980; **17**: 151–161.
9. Sacks T, Moldow CF, Craddock PR, Bowers TK, Jacobs HS. Oxygen radicals mediate endothelial cell damage by complement-stimulated granulocytes. *J Clin Invest* 1978; **61**: 1161–1167.
10. Goldstein IM, Roos D, Kaplan HB, Weissmann G. Complement and immunoglobulins stimulate superoxide production by human leucocytes independently of phagocytosis. *J Clin Invest* 1975; **56**: 1155–1163.
11. Dahinden CA, Fehr G, Hugli TE. Role of cell surface contact in the kinetics of superoxide production by granulocytes. *J Clin Invest* 1983; **72**: 113–121.
12. Fernandez HN, Henson PM, Otani A, Hugli TE. Chemotactic response to human C3a and C5a anaphylatoxins. *J Immunol* 1978; **120**: 109–115.
13. Hugli TE. The structural basis for anaphylatoxin and chemotactic functions of C3a, C4a and C5a. *CRC Critical Reviews in Immunol* 1981; 321–366.
14. Craddock PR, Hammerschmidt D, White JG, Dalmasso AP, Jacob HS. Complement (C5a)-induced granulocyte aggregation *in vitro*. *J Clin Invest* 1977; **60**: 260–264.
15. Löhde E, Kraas E, Abri O, Lierse W. Activated complement—a cause of organ failure in necrotizing pancreatitis. In: Peiper H-J, ed. *Chir. Forum f. experim. u. klinische Forschung*. Heidelberg: Springer-Verlag, 1987: 343–347.
16. Gallin JI, Durocher JR, Kaplan AP. Interaction of leucocyte chemotactic factors with the cell surface. I. Chemotactic factor-induced changes in granulocate surface charge. *J Clin Invest* 1975; **55**: 1049–1057.

ACKNOWLEDGEMENT. The authors wish to thank Mrs Susanne Feldhaus and Mr Klaus Siebert for their excellent technical assistance and Mrs de Bour for preparing the manuscript.

**Received 5 March 1992; accepted 27 March 1992**
